# The Many Faces of Obesity and Its Influence on Breast Cancer Risk

**DOI:** 10.3389/fonc.2019.00765

**Published:** 2019-09-04

**Authors:** Tanya Agurs-Collins, Sharon A. Ross, Barbara K. Dunn

**Affiliations:** ^1^Division of Cancer Control and Population Sciences, National Cancer Institute, Rockville, MD, United States; ^2^Division of Cancer Prevention, National Cancer Institute, Rockville, MD, United States

**Keywords:** adiposity, breast cancer risk, endocrine function, epigenetics, obesity, weight loss

## Abstract

Obesity is associated with increased risk of breast and other cancers. However, the complexity of the underlying mechanisms, together with the interplay of diet and physical activity—contributing to energy balance—and the role of adipose tissue, pose challenges to our understanding of the basis of this increased risk. Epidemiologic studies have documented a higher obesity prevalence in US black women compared to white women. Elucidation of the contribution of potential biological differences among racially distinct groups to their differences in breast cancer (BC) risk and mortality have been topics of considerable interest in recent years. The racial and ethnic variation in body fat distribution may account for at least part of the differences in breast cancer rates in these populations. Yet, while black women exhibit higher rates of obesity compared to white women, this does not translate directly into higher rates of BC. In fact, overall, BC in black women occurs with a lower incidence than BC in white women. Obesity is a known risk factor for postmenopausal breast cancer, and growing evidence suggests that abdominal obesity, also known as central obesity, may increase risk for triple negative breast cancer, which is more common in premenopausal women. The positive association of postmenopausal BC risk and specifically estrogen receptor (ER)-positive BC, is presumably due largely to accumulation of estrogen in the adipose tissue of the breast and other tissues. Of the two main types of adipose tissue—subcutaneous and visceral—visceral adipocytes are more active metabolically. Such adipose tissue harbors multiple molecular entities that promote carcinogenesis: endocrine molecules/hormones, immunologic factors, inflammatory cytokines, metabolic alterations, and other components of the microenvironment. Expression of these culpable entities is largely regulated by epigenetic mechanisms. The interrelationship between these entities and drivers of epigenetic alteration are critical to the regulation of pathways connecting obesity and cancer risk. Initiatives to counteract the carcinogenic effects of obesity have primarily involved modulation of energy balance by diet. However, targeting of specific molecular abnormalities characterizing adiposity offers an alternative approach to preventing cancer. Our goal in this review is to first discuss the major mechanisms contributing to the obesity-breast cancer link. We will also consider race, specifically black/white differences, as they relate to the association of obesity with breast cancer risk. Then we will enumerate strategies targeting these mechanisms to reduce BC risk, in large part by way of dietary interventions with potential to mitigate the cancer-promoting components of adiposity.

## Introduction

Obesity, a state of increased adiposity, is categorized according to body mass index (BMI) as having a BMI >30 kg/m^2^ (1, 2) and is now considered a chronic disease (3). The weight gain, along with associated metabolic disturbances, that characterizes obesity results from disruption of energy balance, causing tissue stress and dysfunction (4, 5). The serious consequences of these physiological effects of obesity have evolved into major health concerns in recent years. Obesity is increasingly becoming a worldwide epidemic, with global obesity rates nearly tripling since 1975 (3). In 2015, the worldwide prevalence of obesity among adults reached 12%, with higher rates among women (2, 6).

## Epidemiology of Obesity and Breast Cancer Risk According to Life Stage and Race

High adiposity (BMI, adult weight gain, and abdominal obesity) is a risk factor for several types of cancer, including breast cancer (7). The association between overweight/obesity and breast cancer risk varies in relation to several factors including menopausal status and specific life stages. For postmenopausal women, several meta-analyses have consistently shown positive associations among high adiposity, adult weight gain, and risk of hormone receptor-positive (estrogen receptor-positive/ER+ and progesterone receptor-positive/PR+) breast cancer (6, 8–12). Conversely, the epidemiologic literature supports an inverse association or no association between high BMI and premenopausal hormone receptor-positive breast cancer risk (13–15). Additionally, high BMI during childhood, adolescence, and early adulthood is associated with decreased risk of premenopausal breast cancer (12, 16, 17). However, the association between measures of adiposity and premenopausal breast cancer risk may vary by ethnicity. For example, a few studies suggest that high adiposity may confer greater risk for premenopausal breast cancer among Asian women (18, 19). Other studies assessed abdominal, i.e., central, adiposity, and found a significantly positive association with both pre-and postmenopausal breast cancer risk (20, 21). The association appears to be strongest with triple negative breast cancer (TNBC), which occurs most often in women under 40 years of age (22). Harris et al. (23) revealed that measures of abdominal obesity (e.g., waist circumference, waist-to-hip ratio) were associated with increased risk for premenopausal ER- breast cancer when examining the highest vs. the lowest quintile for each measurement. Similarly, Pierobon and Frankenfeld (24) demonstrated in a systematic review and meta-analysis that a significant association existed between TNBC and obesity, but when stratified by menopausal status the results were significant only among premenopausal women.

These obesity-breast cancer associations can also be addressed in relation to race or ethnicity. This approach is especially relevant given that the prevalence of obesity in the U.S. is higher among blacks than whites. In 2015–2016, the highest rates of obesity in the U.S. population was among black women (54.8%) (10). This contrasts with an overall rate of 39.8% in the general population. Furthermore, variation in body fat distribution among racial and ethnic groups may account for differences in breast cancer rates by menopausal status and breast cancer subtypes (25–27). However, clear patterns have not been identified. The AMBER Consortium, a collaboration of four studies, examined obesity and body fat distribution among black women (26). In this study, breast cancer subtypes were examined by menopausal status, BMI, and abdominal obesity. For postmenopausal black women, higher recent BMI (> 35 kg/m^2^) was associated with ER+ breast cancer and decreased risk of TNBC. Among premenopausal black women, higher BMI (> 30 kg/m^2^) was associated with decreased risk of ER+ breast cancer. When examining abdominal obesity, breast cancer risk also differed by menopausal status. For postmenopausal black women, a high waist-to-hip ratio (WHR) (>0.88 vs. ≤0.64 cm) was associated with increased risk for each tumor subtype (ER-, ER+, PR-, PR+), and a higher risk for TNBC tumors. In contrast, among premenopausal black women, high WHR (>0.88 vs. ≤0.64 cm) was only associated with increased risk of ER+ breast cancer (26). Other studies have also shown that regardless of menopausal status, abdominal obesity increases the risk for TNBC among black women; TNBC is a particularly aggressive phenotype (22, 27); however, inconsistent results have been reported (28).

The Carolina Breast Cancer Study, which is contained within the AMBER Consortium, demonstrated an increased incidence of TNBC in premenopausal women. An association with obesity is suggested by the observation that women with a high compared to low WHR had a significantly higher risk of developing basal-type TNBC. This increased risk of TNBC in association with obesity applies to both pre- and postmenopausal black women (29), although the risk is highest in premenopausal women (22, 29).

To summarize, the relationship between adiposity and breast cancer risk is complex and varies depending upon several factors. Increased breast cancer risk in postmenopausal women is especially notable among those who are obese (2), as demonstrated in large studies using different study designs (20, 21, 24).

On the one hand, early life obesity is protective against premenopausal breast cancer, whereas the scientific literature provides clear and consistent evidence linking high adult adiposity as a risk factor with postmenopausal breast cancer. Although the incidence of overall breast cancer is lower among black women compared to white women, black women have a higher incidence of ER- and TNBC tumors and their tumors tend to be of a higher grade than tumors in women from other racial and ethnic groups (30). The increased frequency of these tumors may be partially attributable to the higher abdominal adiposity rates in black populations.

### Obesity, Socioeconomic Status, and Breast Cancer Risk

Obesity is associated with socioeconomic status (SES) in high-and-middle income countries (6). In high-income countries, the shift in the food supply created opportunities to consume inexpensive, energy-dense foods with low nutritional value, which is a major driver of the obesity epidemic, especially among low SES individuals (31). For example, a systematic review revealed that lower life course SES was associated with obesity risk (summary OR: 1.35; 95% CI: 1.04, 1.76) and higher waist circumference (summary OR: 4.67; 95% CI: 4.15, 5.20) (32). In women, the overall obesity prevalence was shown to decrease with increased income and educational attainment (33). SES is linked not only to obesity risk, but also to breast cancer incidence and mortality (34). Evidence also exists for a relationship between SES and breast cancer outcomes, with low SES being associated with advanced disease stage at the time of diagnosis, greater disease recurrence, and poorer survival in multiple studies (34). However, other studies suggest that the contribution of SES to racial and ethnic disparities in breast cancer is modest and varies by hormone receptor subtypes and stage at diagnosis (35). Thus, the relationship between SES and obesity may affect breast cancer risk and prognosis differently according to race and ethnicity. Limited research has been conducted to identify a direct association between SES and breast cancer risk (36, 37). However, the indirect link via their mutual association with obesity emphasizes the importance of such investigations, especially in light of the current epidemic of obesity (31).

### Obesity Prevention and Breast Cancer Risk

Intervention studies aimed at reducing the incidence of obesity can provide opportunities to decrease breast cancer risk, specifically post-menopausal breast cancer. The increase in obesity rates is associated with changes in the food and built environments which contribute to increased consumption of energy-dense foods and less physical activity. These changes result in a positive energy balance—the state in which energy intake exceeds energy expenditure—which, over time, can lead to obesity. Several studies have shown that reducing caloric intake and increasing physical activity may be protective against both pre- and post-menopausal breast cancer (38, 39). As such, targeting modifiable risk factors of obesity such as diet and physical activity is one strategy to reduce breast cancer risk and improve survival.

The complex interplay of diet and physical activity, together with the role of adipose tissue, pose challenges to our understanding of the mechanisms by which obesity confers increased breast cancer risk. Furthermore, obesity is intertwined with social deprivation, environmental conditions, genetics, hormones, and epigenetic factors, all of which can impact breast cancer risk and the aggressiveness of breast cancer phenotypes. In this review we discuss obesity and diet-related biological mechanisms with the aim of identifying molecular and behavioral targets that can inform research into novel interventions to reduce breast cancer incidence and mortality. The focus of this review is on the relationship between obesity and postmenopausal breast cancer risk. Although it is an important topic, the interplay between adiposity and breast cancer survival is not addressed here.

## Mechanistic Basis of Obesity and Its Impact on Breast Cancer Risk

### Adipose Tissue as an Endocrine Organ, Regulating Metabolism and Immune Responses

The increased adipose tissue that characterizes the state of obesity is not merely a passive reservoir to store lipids and energy, as once thought. Adipose tissue is biologically active, and is now considered to be an “endocrine organ,” given the multiple factors it produces that impact systemic energy metabolism, neuroendocrine function, and immune responses (40). These areas of adipose function can be broadly classified as protein products that affect the metabolism of distant cells/tissues and enzymes that are involved in steroid hormone metabolism.

### Metabolic Dysregulation in Obesity

In obesity multiple metabolic changes are observed, including alterations in lipids, hyperglycemia and glucose intolerance, and insulin resistance/hyperinsulinemia (1, 5, 41–43). Dysregulated secretion of adipocyte-derived proteins (adipokines) which act both locally and systemically is also observed. These changes in secreted hormones and other factors include increased leptin, decreased adiponectin and resistin, retinol binding protein-4 (RBP4), tumor necrosis factor-α (TNF-α), interleukin-1β (Il-1β), and IL-6 (5, 40, 44, 45). Leptin has been a focus of much early work on obesity. Although the primary function of the protein leptin has generally been viewed as promoting leanness, by signaling back to the CNS to decrease intake of food and increase energy expenditure to limit obesity, the overall role of leptin is far more complex and to date remains somewhat elusive (46). From an oncology perspective, high leptin levels appear to correlate with increased risk of certain cancers, including breast cancer (1).

Of note, all accumulations of adipose tissue, i.e., adipose depots, are not the same. The adipose depots that characterize obesity are complex and must be analyzed at a granular level in order to understand their effect on cancer risk. Excessive visceral deposits of adipose tissue, primarily in the abdomen, are considered to be the main culprits involved in disease causation (47, 48). Specific abdominal organs such as the greater omentum (referred to as the “abdominal policeman”) are preferred sites of this visceral adiposity tissue (VAT). In contrast, subcutaneous adipose tissue (SAT) is generally less active in the mechanisms implicated in these disruptions of biologic homeostasis. Excessive adipose tissue, especially VAT, is associated with the “metabolic syndrome,” involving insulin resistance, hyperglycemia, dyslipidemia, and hypertension. Prothrombotic and proinflammatory states are also characteristic of VAT. Besides the adipocytes, which secrete endocrine hormones such as leptin and adiponectin, adipose tissue contains other types of cells that also secrete proteins. Examples include leukocytes and stromovascular cells which, along with adipocytes, express TNF-α, particularly in SAT (40, 49). These dissimilar cell types function in an integrated manner, consistent with the view that adipose tissue is actually an entire endocrine organ (40). White adipose tissue (WAT), the subtype of adipose tissue whose main function is to store energy in the form of lipids and maintain energy homeostasis (50–52), functions as a complex secretory and endocrine organ. In the obese state adipocytes in WAT secrete a number of inflammatory cytokines, including TNF-α and IL-6 (51).

### Immune Function of Adipose Tissue

These metabolic functions are intimately connected to the immune activities of adipose tissue (4). In addition to adipocytes and stromovascular cells, leukocytes, which include a variety of immune cells—macrophages, neutrophils, T cells, B cells and mast cells—are found in increased numbers in adipose tissue of obese individuals. In particular, macrophages, which make up 5-10% of cells in healthy adipose tissue, constitute 50% of all cell types in hypertrophic adipose tissue (4, 49). The macrophages located within adipose deposits skew toward the M1 type, which secretes inflammatory cytokines, including TNF-α, IL-6, and IL-1β; this contrasts with M2 macrophages which have the antithetical effect of improving metabolic function and reducing adipose inflammation. The inflammatory macrophages are the primary cell type responsible for inflammation associated with obesity. As a result, in obesity the circulating levels of these macrophage-secreted factors are elevated, resulting in a chronic inflammatory state (52). Although self-limited inflammation in response to pathogens is a normal function of the innate immune system, including macrophages, individuals with obesity and metabolic syndrome experience chronic low-grade inflammation, which is associated with higher levels of inflammatory cytokines in both plasma and subcutaneous adipose tissue (4, 53). Such impaired resolution of acute inflammation leads to metabolic tissue stress with tissue destruction and dysfunction (53), including insulin resistance and diabetes (5, 45, 54). Thus, the connection between obesity and metabolic dysfunction/insulin resistance is dependent at least in part on inflammation which is initiated by the innate immune system (54).

The dysfunctional milieu of obesity-associated adipose tissue has additional adverse immune effects, such as ectopic accumulation of lipids in non-adipose tissue, including tissues of the immune system: bone marrow and thymus (49). Obesity results in altered lymphocyte tissue architecture and integrity with shifts in populations of immune cells that lead to inflammatory phenotypes (4). Among these changes are increases in T helper type 1 (Th1) cells and cytotoxic CD8+ T cells, which produce cytokines [interferon-ɤ (IFN-ɤ), TNF, and IL-6] that induce M1 macrophages, which, in turn, secrete proinflammatory cytokines (TNF, IL-6, IL-1β, and others) (49). B cells are also increased in VAT, as shown in mice fed a high-fat diet (48). Total B cells, B-1a cells and B2 cells are all elevated in this setting. Increased abundance of mature B cells which had undergone class switching, including IgG+ cells which are involved in progressive immune activity, is observed. These mice exhibit increased serum concentrations of IgG2c, a pro-inflammatory isotype. B lymphocytes are therefore involved in the development of VAT inflammation, to which they contribute by activating CD8+ and Th1 cells as well as releasing pathogenic antibodies. The downstream metabolic effects of pro-inflammatory cytokine produced by the CD8+ and Th1 cells include insulin resistance and glucose intolerance, which ultimately are attributable to B cell activity.

## Endocrine Function of Adipose Tissue in Obesity Increases Breast Cancer Risk

### Immune System: Role in Breast Cancer Risk

The alterations in the immune system that are associated with obesity can predispose to development of 13 cancer types via a variety of mechanisms (2, 53, 55). The mechanistic underpinnings of the observed causal relationship of obesity with breast cancer exemplify the intertwining of the various adipose mechanisms described above. In one prospective population-based cohort of postmenopausal women followed from 1990 through 2005, 272 women were diagnosed with incident breast cancer. Among three markers altered by obesity [leptin, adiponectin and soluble TNF receptor 2 (sTNF-R2)], plasma levels of sTNF-R2 and leptin showed independent positive association with breast cancer risk (56). Given the known carcinogenic nature of the inflammatory cytokine TNF, derived from macrophages that infiltrate adipose tissue, these data are consistent with an immunologic mechanism linking obesity and breast cancer. In the setting of obesity, WAT becomes altered, manifesting changes in production of steroid hormones and adipokines as well as chronic subclinical inflammation, activities which predispose to cancer (50). M1 macrophages, the CD68 staining immune cells that secrete inflammatory cytokines—TNF-α, IL-6, and IL-1β–that are implicated in promoting obesity-associated inflammation (49), are abundant in breast WAT (50, 52). These macrophages aggregate in histologically defined crown-like structures (CLS) in which they surround necrotic adipocytes, a histopathologic feature that is observed in mice and humans (41, 47, 57). Macrophage-based CLS formations are found in normal breast tissue, at a higher frequency in obese women (58, 59). These breast CLS (CLS-B) serve as measures of breast inflammation, quantified as the CLS-B index (60).

### Steroid Hormones: Role in Breast Cancer Risk

The increased incidence of estrogen-receptor-positive (ER+) breast cancer in obesity supports the role for estrogen, a steroid hormone, in breast carcinogenesis (61), bringing the endocrine function of adipose tissue into play. Key factors that are increased in breast tissue of obese women have been shown to play a role in stimulating expression of aromatase, the enzyme that carries out the rate-limiting step of estrogen biosynthesis (56, 61). The mechanisms responsible for production of these factors rely on activation of the immune system, bridging the previously described immune and hormonal effects of obesity. For example, TNF produced by adipose-infiltrating macrophages stimulates expression of aromatase in adipose fibroblasts (56, 61). Prostaglandin E_2_ (PGE_2_), an inflammatory factor, and hypoxia-inducible factor 1α (HIF-1 α) both participate in inducing aromatase production by adipose stromal cells (ASCs) (62). Elevated levels of aromatase are found in VAT and SAT as well as adipose tissue in the breast of obese postmenopausal women (63), including inflamed breast adipose tissue of obese women with breast cancer (64). This “obesity-inflammation-aromatase axis” has been proposed to play an important role in increased risk of ER+ breast cancer in postmenopausal women, by elevating estrogen levels in the breasts of women in whom levels of estrogen in the general circulation are reduced (60, 64, 65).

## Molecular Mechanisms Contributing to Obesity and Breast Cancer: Genetics, Epigenetics, and Microbiomics

At the molecular, mechanistic level, genetics, epigenetics, and microbiomics are likely involved in susceptibility to weight gain and obesity (66). These molecular factors may also interact to give rise to obese phenotypes. Furthermore, the interaction between these molecular factors with behavior and environmental factors likely add to the etiologic complexity and biological variation that is observed with weight gain and the obese state. Moreover, dysregulation of these molecular mechanisms may explain not only the link between obesity and breast cancer, but also the comorbid conditions associated with obesity.

### Genetics

Many gene variants have been found to be associated with obesity. Recent reviews highlight both the candidate gene approach utility for identifying monogenic obesity genes as well as genetic variants identified through Genome Wide Association Studies (GWAS), which implicate genes from several biological pathways in polygenic obesity (66–68). These GWAS approaches have revealed that loci associated with obesity carry genes involved in pathways influencing neuro-circuits of appetite and satiety regulation (*BDNF, MC4R, NEGR, POMC*) (69–73), insulin secretion and action (*TCF7L2, IRS1*) (69, 74), adipogenesis (75) and energy and lipid metabolism [*FTO, RPTOR, MAP2K5* (69, 74, 76)]. Using well-powered GWAS studies, more than 870 SNPs have been found to be associated with BMI (68). However, the findings also indicate that these loci only explain 5% of the variance of BMI (77). Although challenging, attempting to explain the remaining variability is a focus of obesity research. In this regard, the utilization of other omics, such as transcriptomics, proteomics, epigenomics, microbiomics, and metabolomics, may increase the phenotypic prediction of weight gain (66, 78). Associations between obesity, genetics and breast cancer have been documented and more are emerging. One example concerns the fat mass and obesity associated (*FTO*) gene, which was the topic of a recent systematic review that promulgated *FTO* gene as a possible mediator for the association between obesity and breast cancer (79). The *FTO* gene encodes a dependent oxygenase related to 2-oxoglutarate that has a role in DNA demethylation but its molecular mechanism in obesity and metabolism has not been elucidated (80). In their systematic review, Akbari et al. (79) suggested that polymorphisms in the *FTO* gene may influence the risk of breast cancer as well as obesity through expression of the homeobox transcription factor iriquois 3 (*IRX3*) gene. *IRX3* is a developmental transcription factor that more recently has been implicated in regulating energy expenditure (81).

### Epigenetics

With a great degree of complexity and flexibility, epigenetic mechanisms influence how genetic information is transcribed and translated into proteins, ultimately affecting health and disease, including the conditions of weight gain and obesity. In contrast to genetic modifications, which lead to a change in the base sequence of DNA, epigenetic changes are thought to be reversible and consist of chemical modifications to DNA (or DNA-associated chromosomal proteins called histones) that occur in the absence of a change in the DNA sequence (82). Epigenetic mechanisms include DNA methylation, histone modifications, and microRNA-mediated regulation, which can be passed on mitotically (through cell division) or meiotically (through generational inheritance) (83). Epigenetics has emerged as a significant link between genes and the environment, serving as a molecular mechanism to explain individual variation in biological response to environmental factors. Interestingly, recent evidence suggests an association between obesity and DNA methylation; but whether this is a cause or a consequence of the obese phenotype requires mechanistic examination (84). A brief discussion of the relationship between DNA methylation, obesity and breast cancer follows. The role of microRNA and histones in influencing obesity and their relationships to breast cancer are discussed elsewhere (83, 85–87).

#### DNA Methylation

In mammals, the addition of methyl groups to DNA (methylation) occurs predominantly at cytosines adjacent to guanines (“CpG” sites) through DNA methyltransferases. Promoter DNA methylation disrupts the binding of transcription factors and attracts methyl-binding proteins that typically initiate chromatin compaction and gene silencing (88). Promoter hypomethylation, on the other hand, is associated with activation of transcription. DNA methylation is the best studied and most stable epigenetic mechanism, and both candidate gene methylation and epigenome-wide methylation studies have been performed to understand connections with obesity (68, 83, 87). These have led to discovery of DNA methylation changes that are associated with many genes and pathways related to obesity and its comorbidities, including appetite control, insulin signaling, immunity, and inflammation. Interestingly, candidate genes implicated in monogenic obesity (e.g., *POMC*) have also been found to be influenced by DNA methylation changes contributing to common obesity (89). With the use of genetic association analyses along with epigenome-wide association analyses, alterations in DNA methylation have been shown to be the result of obesity rather than the cause of obesity (90). This study suggested epigenetics as a mechanism by which some individuals with excess BMI move to the next step in the causal pathway to metabolic disease. Other evidence, however, is suggestive of a putative causal relationship for DNA methylation alterations in the onset of obesity and metabolic disease. Such is the case for evidence from the Dutch Winter Hunger cohort with inclusion of subjects that experienced famine early in life (91). Investigators recently performed a genome-wide analysis of differential DNA methylation in whole blood from this cohort (92). They show that the associations between exposure to an adverse environment during early development and health outcomes in adulthood are mediated by alterations in DNA methylation; interestingly, *PIM3* methylation (cg09349128), a gene involved in energy metabolism, mediated approximately 13% of the association between famine exposure and BMI.

#### Obesity, Epigenetics, Breast Cancer

There is an emerging interest in interrogating DNA methylation as a possible mechanistic link between obesity and breast cancer. An example concerns estrogen receptor 1 (*ESR1*) gene hypermethylation, which may be involved in the development of breast cancer. Investigators hypothesized that BMI and estrogen-related reproductive risk factors may influence the methylation status of the *ESR1* CpG loci in the normal breasts of healthy women. They found that *ESR1* promoter methylation in women with a BMI ≥30 kg/m^2^ was higher than in the subgroups of women with BMI <25 kg/m^2^ or BMI 25–29 kg/m^2^ and was also higher in postmenopausal women compared with that in premenopausal women (93). The finding provides possible clues to the relationship between epigenetic changes within the *ESR1* gene CpG island and postmenopausal obesity and aging in cancer-free women, and merits additional study. In another example, investigators explored the association of adiposity-related CpG loci and subsequent risk of postmenopausal breast cancer, colorectal cancer and myocardial infarction (94). Using peripheral blood leucocytes from over 1900 individuals from four prospective European cohorts, these investigators measured the relationship between DNA methylation profiles and body mass index, waist circumference, waist-hip and waist-height ratio within a meta-analytical framework that also assessed the relationship of adiposity-related CpG to comorbidities. Among the 40 adiposity-related CpG loci identified, two loci in *IL2RB* and *FGF18* and one CpG locus in an intergenic region of chromosome 1 were associated with colorectal cancer and myocardial infarction development (94). However, none of the adiposity-related CpG loci were associated with post-menopausal breast cancer following Bonferroni correction; the authors also noted that the number of post-menopausal breast cancer cases included in the study was relatively small.

DNA methylation has been suggested as a mechanism that could explain inter-individual variability in terms of weight loss response as well as the metabolic response to weight loss (95). In this regard, there is interest in examining whether weight loss might reverse abnormal DNA methylation changes observed in obesity and thereby reduce comorbidities. Rossi et al. identified several hypermethylated gene promoters in mice that were obese, compared to leaner controls (96). Interestingly, many of these genes showed intermediate methylation in formerly obese mice, suggesting that some obesity-associated epigenetic changes may be resistant to reprogramming after weight loss. These authors also found that weight loss in the formerly obese mice did not reduce proinflammatory cytokine gene expression nor the basal-like mammary tumor burden (96). The authors mention that weight loss in combination with epigenetic or anti-inflammatory interventions may be needed to disrupt the obesity–breast cancer link. Furthermore, examination of DNA methylation, in combination with genetic variants, gut microbiota and other molecular mechanisms, might be useful in understanding the relationship between obesity, weight loss and breast cancer.

### Microbiomics

The collective genomes of the microbes (composed of bacteria, bacteriophage, fungi, protozoa, and viruses) that live inside and on the human body are referred to as the microbiome (97). Alterations of gut microbiota and its microbiome are associated with obesity and are responsive to weight loss (98). For example, transferring the luminal contents from the ceca of obese and lean mice to germ-free animal recipients resulted in more weight gain over a 2-week period in recipients receiving the microbes from obese animals compared to the recipients inoculated with the lean mouse microbes, despite equivalent food intake (99). Hints are also found from human studies, including a study in twins which found that obese individuals displayed reduced bacterial diversity, a depletion of *Bacteroidetes* as well as greater abundance of carbohydrate and lipid-utilizing microbial genes compared to lean individuals (100). Many mechanisms have been implicated in these associations such as increased dietary energy harvest, microbe-induced changes in host glucose and lipid metabolism, microbial signaling through host endocrine systems, and chronic low-grade inflammation leading to insulin resistance (98). Backhed et al. observed a direct link between the intestinal microbiome and increased adiposity when they inoculated germ-free mice with the cecal contents from conventional mice (101). These recipient mice gained weight despite calorie restriction; experiments revealed that weight gain was in part due to increased intestinal monosaccharide absorption and increased hepatic lipogenesis. Furthermore, the microbiome in these mice suppressed a host gene (*Fiaf* or fasting-induced adipose factor) coding a circulating lipoprotein lipase inhibitor (Angptl4), which resulted in an increase in triglyceride deposition in adipose tissue (102). The magnitude of the contribution of the gut microbiota and its gene content to obesity and its related comorbidities is still uncertain (66). Perhaps a better understanding of host-microbe and microbe-microbe interactions may lead to the development of novel strategies for reversing obesity (103).

Microbial perturbations (dysbiosis) have been observed in breast cancer patients compared to healthy individuals (104, 105). Here it is interesting to note that the gut microbiota may influence the production of estrogen metabolites and it has been hypothesized that alterations in the microbiota might lead to elevated levels of circulating estrogens and its metabolites, thus increasing the risk of breast cancer (105). Although an altered intestinal microbiome has been implicated in obesity and alterations of the microbiome (both distal and local) may influence breast cancer risk, little to no research has examined the mechanisms that may explain the association between obesity, microbiome and breast cancer.

## Tackling Obesity: The Many Facets of Weight Loss

### Obesity-Targeting Weight Loss Interventions—Addressing Above Mechanisms

Several observational studies found that adult weight loss was associated with decreased risk for postmenopausal breast cancer (106–109), although others did not find an association (110, 111). A meta-analysis assessing the effect of weight loss on breast cancer incidence found that weight loss significantly reduces breast cancer risk in both pre- and post- menopausal women (112). In a recent study, investigators examined the effect of weight change on breast cancer incidence in 61,335 postmenopausal women enrolled in the Women's Health Initiative Observational Study (109). This study reported that women who lost weight (> 5% of body weight) compared to women with stable weight had a significantly lower breast cancer risk (HR, 0.88, 95% CI, 0.78–0.98). Similar findings were found in the Nurses' Health Study for weight loss and reduced breast cancer risk (HR, 0.77, 95% CI = 0.65–0.91) (108). These results are also supported by bariatric surgery research revealing a reduction in the risk of breast cancer (113). Although the presented evidence that weight loss is associated with decreased breast cancer risk appears to be convincing, more rigorous data involving clinical trials and timing of weight loss are needed.

Weight loss, a state of negative energy balance, is believed to significantly influence postmenopausal breast cancer risk through alterations in several pathways including sex-steroid hormones, endocrine hormones, and inflammatory markers. Obesity-targeting weight loss interventions that include hypocaloric diets and/or exercise have been shown to significantly reduce total body weight, adipose tissue (visceral and subcutaneous) and biomarkers associated with breast cancer risk (114). Here we review how weight loss can modulate obesity-related mechanisms that favor decreased breast cancer risk. Randomized trials of weight loss as an intervention in cancer survivors has been reviewed elsewhere (115, 116).

### Weight-Loss and Sex-Steroid Hormones

As described above, excess adipose tissue modulates steroid aromatization, resulting in elevated levels of estrogen and, therefore, increased breast cancer risk. Weight loss interventions have been shown to have beneficial effects on estradiol, free estradiol, sex hormone binding globin (SHBG) and free testosterone concentrations (117, 118). For example, the Nutrition and Exercise in Women (NEW) study revealed that participants in the diet plus exercise group had greater reductions in total body weight and waist circumference compared to diet-only and exercise-only groups (mean 8.9, 7.2, 2.0 kg, respectively) (119). A dose-response relationship was also found, such that greater weight loss was associated with greater decreases in estrone, estradiol, free estradiol, and free testosterone, as well as a greater increase in SHBG (120). Another study found that overweight and obese postmenopausal women with >10 vs. <10% weight loss, had significant changes in bioavailable estradiol (*p* < 0.001), testosterone (*p* = 0.033), and SHBG (*p* < 0.001) (121). Research studies and meta-analyses provide sufficient evidence that weight loss interventions, in the form of reduced caloric intake and exercise, are associated with significant reductions in sex-steroid hormones (39, 118).

### Weight-Loss and Endocrine Hormones (Insulin and IGF-1)

Abdominal obesity, specifically visceral fat, is associated with metabolic abnormalities such as hyperinsulinemia, insulin resistance and elevated IGF-1 concentrations, all of which are risk factors for breast cancer (122, 123). Obesity-targeting weight loss interventions have produced favorable changes in fasting insulin, glucose and HOMA-IR concentrations (121, 124–126). For example, weight losses > 10% were associated with a median absolute change in insulin concentrations (−3.4 μIU/ml; *p* = 0.018) among women at increased risk for breast cancer (121). Another study revealed that weight loss (subcutaneous and visceral fat) at 6 months was significantly associated with reductions in fasting insulin and HOMA concentrations, which remained significantly lower than baseline at 12 months, even after weight regain for women assigned to the diet group (124). However, the literature is somewhat contradictory as it relates to insulin-like growth factor-1 (IGF-1) concentrations. Several weight loss interventions have shown that weight loss is positively associated with IGF-1 concentrations and decreased IGFBP-1 & 3 (114, 121, 124, 127). Mason et al. (128) found no significant changes in IGF-1 or IGFBP-3 by intervention arm, but did find that greater weight loss was associated with elevated IGF-1 and molar ratio of IGF-1: IGFBP-1 concentrations in obese postmenopausal women. However, a few interventions found either no significant change (125) or slight decreased serum IGF-1 and increased IGFBP-3 concentrations after the adoption of a very low-calorie diet (129). A multicenter trial examining caloric restriction of 25% over 2 years suggests that insignificant changes in IGF-1 and IGF-1:IGFBP-3 molar ratio concentrations may be related to chronic high protein intake (130). It is well-established that weight loss can reduce insulin, glucose, and measures of insulin resistance. However, large intervention studies are needed to better understand the effects of weight loss on IGF-1 concentrations.

### Weight Loss and Inflammatory Markers

White adipose tissue is metabolically active and is a major contributor to the release of cytokines and adipokines in the bloodstream (131). Weight loss interventions have shown reductions in systemic markers of chronic inflammation (121, 124, 132–134). A study in obese postmenopausal women found that those randomized to the diet plus exercise group and the diet only group experienced the greatest amount of weight loss, which, in turn, was associated with significant increases in adiponectin (+11.7 % and 18.5% in each group, respectively) as well as reductions in leptin (*p*-trend < 0.001), compared to the control group (135). Another study found that obese postmenopausal women assigned to a hypocaloric diet plus aerobic exercise condition vs. a diet-only condition lost more weight, particularly abdominal fat, and had significantly greater reductions in C-reactive protein (CRP), IL-6, sIL-6R, and TNFR1 concentrations (136). In this study, reductions in abdominal fat stimulated lipolysis, which correlated with reductions in plasma IL-6 and TNFR1 (136). Other studies in obese postmenopausal women reported that > 10% total weight loss and reductions in waist circumference produced favorable changes in CRP, adiponectin, leptin, and the molar ratio of adiponectin: leptin concentrations at 12 weeks and at 1- year follow-up (121). A systematic review and meta-analysis found that diet-induced weight loss was associated with reductions in adiponectin concentrations (137). Similar findings have shown reductions in several systemic concentrations of acute phase reactants and pro-inflammatory cytokines after weight loss intervention (124, 138, 139). Nicklas et al. (140) observed that the strongest correlations with change in CRP was a change in weight, waist circumference, insulin and HOMA. Overall, obesity-targeting weight loss interventions have shown reductions in most inflammatory markers, especially for CRP.

### Weight-Loss and Macronutrient Composition

There may be differential amounts of weight loss in response to specific dietary macronutrient (e.g., protein, fat, carbohydrate) composition. Several meta-analyses of weight loss randomized controlled trials (RCTs) examined the efficacy of low-carbohydrate (LC) vs. low-fat (LF) diets on weight change (141–143). One study found non-significant differences for macronutrient composition on the amount of weight loss at 12 months (144); whereas, the other meta-analyses found that LC diets rather than LF diets led to significantly greater weight loss at 12 months, but the weight loss differences between diets were small (141–143). Additionally, two large RCTs did not observe differential effects of macronutrient intakes on the amount of weight loss (145, 146). Specifically, the POUNDS LOST trial did not find differences in 4 diets that varied in macronutrient composition on changes in body composition, abdominal fat, or hepatic fat (145). The DIETFIT study examined the effects of a healthy LF vs. a healthy LC diet on weight change in 609 overweight participants. There were no significant differences between the two diets in terms of weight loss (−5.3 kg HLF and −6.0 kg HLC) nor were there between-group differences for BMI, body fat percent, or waist circumference at 12 months (146). It appears that a reduction in total energy intake may be more important for weight loss rather than manipulating the macronutrient content of the diet. However, the literature is mixed, and further study is required.

### Weight-Loss, Macronutrient Composition, and Biomarkers

Fasting glucose and insulin may impact response to weight loss diets with different macronutrient composition. Researchers suggest that a LC diet may provide greater weight loss in overweight and obese women who are insulin resistant (147, 148); in contrast, normoglycemic participants lose more weight on an LF diet (149). A recent study found that overweight/obese participants who were insulin resistant (HOMA-IR >4) lost significantly more weight on a high-fat (HF) high-protein (HP) diet; however, it should be noted the diet was also very low in carbohydrates (40% fat, 25% protein, 35% carbohydrates) compared to a HF-average protein diet (40% fat, 15% protein, 45% carbohydrate) (149). Rock et al. (133) found that women who were insulin sensitive lost greater weight at 12 months in the LF vs. LC diet group. However, a large RCT did not reveal differential effects for the LF vs. LC diets on weight loss by baseline insulin status (146, 150). Our understanding of macronutrient composition on weight loss in obese insulin-sensitive and insulin resistant individuals requires further study.

Furthermore, it is possible that there is a differential weight loss response to diet composition and that biomarkers associated with breast cancer risk may mediate this association. For example, a LC vs. LF weight loss diet was associated with increased adiponectin concentrations in obese women; however, there were no correlations between weight loss and increased adiponectin (151). Other studies did not find significant differences by intervention arm (caloric-restricted LF vs. LC diet) on favorable changes in adipokine and leptin concentrations at study completion, although leptin concentrations decreased with both diets (152). Weight loss induced by overall caloric restriction rather than the macronutrient content of the diet appears to be more effective in reducing chronic systemic inflammation (140, 153–155) and endocrine markers such as insulin and HOMA (156). Research is needed using large RCTs to understand whether differential weight loss response to macronutrient composition is influenced by biomarkers of breast cancer risk.

### Pharmacological Approaches to Obesity and Weight Loss

Although our emphasis has been on weight loss as a remedy to obesity, other approaches are being tried. As previously discussed, increased physical activity has potential to decrease breast cancer risk, at least in part by reducing obesity (39). However, targeting physical activity as an isolated behavioral change whose increase might facilitate decreased obesity is complicated by the interplay between this approach, caloric reduction and their effects on energy balance. Alternatively, pharmacological approaches to weight, and hence obesity, reduction have been considered. Metformin, the first-line treatment for type II diabetes, which has been extensively studied regarding its cancer preventive activity, including breast cancer (157), has exhibited efficacy in reducing weight in a number of studies. Weight reduction is expected to disrupt the association between obesity and cancer, suggesting a possible mechanistic basis for the anti-cancer effect of metformin (158). In a study of 154 consecutive non-diabetic, overweight/obese individuals, metformin-treated patients had a mean weight loss of 5.8 ± 7.0 kg in contrast to a loss of 0.8 ± 3.5 kg in an untreated group (159). A meta-analysis of 13 studies addressing the effects of metformin on simple obesity showed that metformin is effective in reducing body weight in this population, without inducing hypoglycemia (160). The Diabetes Prevention Program is a clinical trial that randomized 3234 participants with elevated glucose and overweight/obesity, to metformin, intensive lifestyle intervention (ILS), or placebo. Whereas, at 1-year follow-up, only 28.5% of participants in the metformin arm had lost at least 5% of their weight, 62.6% in the ILS group and 13.4% in the placebo group had achieved this goal (161). In contrast, between years 6 and 15, after unmasking, maintenance of mean weight loss was 6.2% with metformin, 3.7% with ILS, and 2.8% with placebo, suggesting a benefit to metformin with respect to a long-term weight loss endpoint. Although much remains to be investigated, metformin has exhibited potential to induce weight loss in both diabetic and non-diabetic individuals.

Another agent showing benefits for weight management is liraglutide, a glucagon like peptide-1 (GLP-1) receptor agonist that is approved by the Food and Drug Administration (FDA) as an adjunct to diet and exercise for management of type 2 diabetes. A review of five randomized clinical trials showed that compared to placebo, liraglutide was associated with a higher proportion of patients achieving at least a 5-10% weight loss (162). The main drawbacks to its use are gastrointestinal side effects and the need for injection. In addition, pharmacologic agents that have been investigated for treatment of eating disorders also offer possible interventions to induce weight loss in obese patients (163). One such agent, lisdexamfetamine, a central nervous system amphetamine, has been used in children with severe obesity, although long-term use is discouraged, given its high potential for abuse (164). The state of pharmacologic interventions to induce weight loss thus remains in flux as studies aimed at identifying an improved balance between efficacy and side effects continue.

## Conclusions and Future Directions

Obesity has reached epidemic proportions in the United States and increasingly around the world. Undesirable health-related sequelae are expected to follow as the obese state is increasingly being observed in children and young adults. Obesity is physiologically complex, however, and we have discussed only a few of the endocrine, immunologic and molecular abnormalities that characterize this state. In addressing the need for reducing obesity we have concentrated on evidence derived from weight loss initiatives. However, other approaches are currently being undertaken. For example, physical activity as a major intervention, with or without accompanying diet directives, has potential to improve obesity-related metabolic parameters. Intermittent fasting approaches, including time-restricted feeding, are emerging weight loss strategies, which may also improve metabolic parameters. Bioactive food components such as omega-3-fatty acids are being studied as interventions to facilitate loss of weight. Finally, pharmacologic approaches, including agents such as metformin, need to be investigated in relation to their weight reducing efficacy.

Breast cancer, the most common cancer in postmenopausal women in the U.S., is one of the malignant outcomes associated with chronic obesity. Thus, efforts to improve interventions to prevent breast cancer, along with other serious obesity-associated diseases, require a deeper understanding of the physiological basis of obesity as well as the development of interventions to reduce this high-risk state in the population.

Despite the extensive research that has been ongoing into the multiple facets of obesity on general health and cancer in particular, huge gaps remain in our understanding of mechanisms and associations. Of immediate interest is the disconnect between obesity's positive association with postmenopausal ER-positive breast cancer and its inverse association with premenopausal ER-positive disease; what is the mechanistic basis for this difference? How do the duration and timing in the life cycle influence the chronic nature of obesity that appears to be linked to breast cancer? Additional gaps address the complex molecular mechanisms at the genetic and epigenetic levels which control expression of proteins that contribute to obesity. The integration of various omics data, including transcriptomics, proteomics, epigenomics, microbiomics, and metabolomics, may also assist in elucidating the link between obesity and cancer.

The majority of the epidemiologic studies linking obesity to breast cancer used self-reported anthropometric measures (e.g., BMI, waist circumference) to assess risk. However, more meaningful assessments of body composition compartments (e.g., VAT and SAT), which capture known physiological and metabolic changes associated with breast cancer risk, need to be used in future studies. Also, one must not ignore the enormous effect the obesity epidemic is having on low SES populations, which in the future may potentially lead to associated chronic diseases, including cancer. Lastly, since the majority of the studies were conducted among Caucasian women, research is needed to understand the association between body fat distribution and specific breast cancer subtypes across various racial and ethnic groups.

## Author Contributions

TA-C, SR, and BD each wrote sections of the manuscript and each reviewed and edited the manuscript for content and cohesion.

### Conflict of Interest Statement

TA-C, SR, and BD are employees of the National Cancer Institute, National Institutes of Health in Rockville, MD.
